# Numerical simulations of heat generation, thermal radiation and thermal transport in water-based nanoparticles: OHAM study

**DOI:** 10.1038/s41598-023-42582-4

**Published:** 2023-09-20

**Authors:** Farwa Waseem, Muhammad Sohail, Showkat Ahmad Lone, Gilbert Chambashi

**Affiliations:** 1 Department of Mathematics, Khwaja Fareed University of Engineering and Information Technology, Rahim Yar Khan, 64200 Pakistan; 2 Department of Basic Sciences, College of Science and Theoretical Studies, Saudi Electronic University, Riyadh, 11673 Saudi Arabia; 3 School of Business Studies, Unicaf University, Longacres, Lusaka, Zambia

**Keywords:** Energy science and technology, Mathematics and computing, Nanoscience and technology

## Abstract

This study investigates the 3D flow properties and heat transfer of copper, titanium/ water nanofluids across a bidirectional surface under the impact of MHD. The thermophysical features of nanofluid are employed using the Tiwari and Das model. Boundary layer theory has simplified the resulting physical principles. By using the proper transformations, the complicated sets of connected PDEs have evolved into ODEs. Equations that have been modify by using OHAM. For various dimensionless component ranges between $2\le M\le 10$.$0\le a\le 3$, $0.05\le \varphi \le 0.08$, $3\le \mathrm{Pr}\le 8$, $0\le Rd\le 6$, and $2\le \lambda \le 4$ the results are investigated computationally and graphically. It is observed that fluid parameters improve; they react differently from temperature and velocity profile. Additionally, thermal profiles decrease in comparison to greater Eckert and Prandtl numbers.

## Introduction

Many industrially used mechanisms have important uses in thermal transportation. Nanoparticles are a popular area of study right now. Due to the numerous applications of nanoparticles, researchers have thoroughly examined their inclusion in various combinations. The copper–water nanofluid in a hollow, for instance, was explored by Marzougui et al.^1^. They examined the physical features and effects of a number of associated parameters using the COMSOL tool. Additionally, they published the results of the mathematical investigation of entropy production. Mahian et al.^2^ examined the importance of modelling and simulation of nanofluid flow. The discussion emphasized the high expenses associated with conducting experimental studies for various applications such as photovoltaic liquid warming systems and electrical gadget chilling. Experimental analysis of the effectiveness of multilayer films was done by Bhutta et al.^3^. They utilized the amalgamation process. In situ PI polymerization on a carbonized nanofiber (CNF) framework is used by Zhao et al.^4^ to create a free-standing PI electrode. The CNF framework serves as a current collector and conductive channel for electronic devices. Arshad et al.^5^ conducted a study involving two distinct hnf flows between two parallel plates positioned at varying heights. Their investigation led them to the conclusion that hybrid nanofluids containing oxide nanoparticles exhibit greater efficiency in comparison to hybrid nanofluids containing a mixture of nanoparticles. Li et al.^6^ addressed the influence of thermal and solutal Marangoni convections on the transport characteristics within a Casson hybrid nanofluid flow passing through a rotating disk. Madhukesh et al.^7^ conducted an examination of the thermal efficiency of a (thnf) within a accessible inclined cylinder/plate combination. Their research highlighted the increasing need for energy-efficient cooling devices across diverse manufacturing and energy associated sectors. Sreenivasa et al.^8^ investigated the impact of chemical reactions and heat source/sink upon the flow of (hnf) across a stretching cylinder. They considered the existence of a magnetic dipole and a porous media. Yang et al.^9^ investigated the extension of corona resistance lifetime exhibited in high-temperature circumstances for PI materials loaded with a specific ratio (2wt%) of nanoparticle Al_2_O_3_. The "thermal stabilization effect" is proposed as a possible explanation for this unexpected phenomenon, with an emphasis on the exciton aiding in completion using the pulsed electroacoustic (PEA) method. Thin Nano films called PI nanocomposites have complicated chemical structures that enable them to achieve sample thicknesses of 100 m or greater. The process of creating PI/SiO_2_ is complicated and involves a lot of variables. According to Akram et al.^10^, a thorough synthesis procedure is provided for creating PI and PI/SiO_2_ as well as method optimization for PI/SiO_2_ layers. Lia et al.^11^ have examined the mentioned occurrence by incorporating the effects of buoyancy and viscous dissipation within the (hnf). Adelmalek et al.^12^ investigated hybrid nanoparticles in Carreau fluid using chemical reactions and heat generation as inspirations. The Galerkin strategy was employed to control the nonlinear transformed differential equation system. Vaidya et al.^13^ examined of the mixed convection phenomenon in nanofluid passing across a nonlinearly stretched Riga plate. They considered both the buoyancy-assisting and opposing region during their exploration. In the research carried out by Arshad et al.^14^, an examination was undertaken concerning the heat and mass transfer happening across unstable infinite porous exterior, while considering the influence of chemical reactions. Nazir et al.^15^ explored the use of two viscosity models to study heat and momentum transmission in a spinning Carreau fluid as it flows through a cone. Alsulami et al.^16^ studied heat transfer in a non-Newtonian fluid containing Ti6Al4V and AA7075 nanoparticles in a porous medium under local thermal non-equilibrium constraints. Mansour et al.^17^ explored the numerical analysis of MHD mixed convection in a rectangular cover chamber containing stretching cylinder microorganisms. The left side of the rubber gasket hollow is rising vertically at a consistent rate despite various magnetic field inclinations. Benos et al.^18^ addressed the essential influence of aggregations on the natural convection of Carbon nanotube-water nanofluid in MHD. Akbar and Sohail^19^ performed an analysis on the 3-D magnetohydrodynamic viscous flow, considering the inspiration of thermal radiation and viscous dissipation. They focused on the behavior of the flow as it moves over a horizontally stretched nonlinear surface situated in two perpendicular directions. In their study, Arshad et al.^20^ examined the 3-D MHD nanofluid flow involving chemical reactions and thermal radiation over a double stretching surface, under the inspiration of an inclined magnetic field. Their findings indicated that the highest level of heat transfer occurs when thermal radiation is inattentive and the slanting magnetic field aligns parallel to the axis of rotation. Shahzadi and Nadeem^21^ worked on the magnetohydrodynamic analysis of nanofluid flowing through a permeable medium. In a water model based on chemical reactivity, Shah et al.^22^ examined the behavior of nanoparticles. They talked about how a stretching sheet affected the flow dynamics. They performed a comparative analysis to evaluate the validity of the suggested plan and the solution and found that the latter is strongly supported by the former. Lin and Yang^23^ considered the nanofluid flow happens in broad applications, and subsequently has gotten far reaching consideration. Due to the variations in heat and pressure transfer between two types of flowing, the transition of nanofluids from one to the other is a crucial issue. When nanofluids leave the thermal equilibrium or dynamic equilibrium state, they will become unstable. Using the Cattaneo-Christov theory, Nawaz et al.^24^ studied heat transfer in a hybrid nanoparticle combination. They take into account the irregular magnetic field. Through the use of FEM, they handled the flow of the expressions. Sheikholeslami et al.^25^ studied a polarizable hybrid nanofluid of MWCNT-Fe_3_O_4_/H_2_O in a spherical chamber containing two rotating heaters. Nisar et al.^26^ analyzed dispersion in compressed flow behavior using a hybrid model between rotating discs. They used stream line patterns to illustrate the grip parameter's dynamic behavior. When compared to the radiation parameter, they noticed a shift in the thermal profile. Ali^27^ conducts an empirical examination of the inner convective transport of SiO_2_/water nanofluids in a pipe under a totally stormy environment. We measured the convective heat transfer coefficients of the nanofluid and the Nusselt number for three distinct volumetric quantities of nanoparticles (0.001, 0.003, and 0.007%). At several locations along the tube with changing Reynolds numbers, local convective coefficients for heat transfer were also measured. Waqas et al.^28^ studied of Burger Bio convective nanofluid containing gyrotactic bacteria examined the role of activation energy in heat transfer. They used the slip theory to develop the boundary conditions. For transformed nonlinear equations, the built-in BVP4C module in MATLAB is used to estimate the solution. Haq et al.^29^ focused at the flow characteristics of Cross nanoparticles over wide areas as a result of the magnetism and Arrhenius excitation potential. Ahmad et al.^30^ conducted an analyzed of a bioconvection nanofluid flow through a stretching sheet in a permeable medium containing gyrotactic motile bacteria and allowing a chemical reaction. Sohail et al.^31^ explored at the significance of temperature-dependent thermal conductivity and coefficient of diffusion for mass and thermal transmission in the Sutterby model using OHAM. Aldabesh et al.^32^ studied nanofluid movement across rotating discs, considering Brownian diffusion and thermophoresis. Researchers found larger Biot numbers enhance the concentration field. The fundamental rheological properties of “Jeffrey's gyrotactic motile microorganism” like nanofluid were quickly advanced by Khan et al.^33^. Dawar et al.^34^ explored impact of momentum slip in the movement of micropolar fluid. In a numerical investigation, Khan et al.^35^ looked at the structural evaluation for nanofluid transport along curved channels. Javed et al.^36^ investigated how chemical processes affected peristaltic flow in a curved, wave-like channel when slip circumstances were present. Using a finite element method, Slimani et al.^37^ investigated convective heat transfer in a hybrid nanofluid model in a permeable containment. Shahzad et al.^38^ conducted an explored focused on the dynamics of axisymmetric motion occurring on a radially extending surface. The productivity of photovoltaic was studied by Sheikholeslami et al.^39^ as part of an improvement in thermal energy connected to flat plate solar collectors. Overviews of crucial economic systems and solar water heating were presented. Sheikholeslami et al.^40^ investigated how nanoparticle thermal properties behaved, specifically toward solar collector plates. Along with simulating nanoparticles, they also emulated entropy formation. Recently, some additional researchers have made an effort to analyze the dynamics of stretch flow as mentioned in References^41,42^. Kumbhakar and Nandi^43^ investigated the properties of unsteady (MHD) permitted convective (hnf) through heat transfer to a vertical plate with oscillations. The research considers the impact of thermal radiation and internal heat generation on the boundary temperature and other factors escalating at the boundary. Nandi et al.^44^ studied the impact of heat generation and nonlinear thermal radiation on the flow of CH_3_OH -based nanofluid over a permeable stretching sheet in a porous domain. The fields of temperature and conserving procedures, aerospace, paints, medications, beauty products, and other fields may all significantly benefit from the work presented here.

The previously mentioned studies have concentrated on the analysis of thermal and mass transport utilizing the conventional nanofluid model. In this research, we expand upon the work carried out by Khan et al.^45^, incorporating the well-known Das and Tiwari nanofluid model. This extension involves the consideration of distinct shape factors alongside the influence of magneto-hydrodynamics. Our work introduces a formulation for the diffusion of copper and titanium dioxide nanoparticles within a base fluid, which is water, using a model that accounts for the impact of a magnetic field and the Joule heating phenomenon. Furthermore, we analysis different forms of nanoparticle as mentioned in Table 2. The conducted research is structured as follows: Section “Introduction” comprises the introduction, while Section “Model features and numerical modeling” presents the model features and numerical modeling. Section “Optimal homotopy analysis method” analysis the solution methodology, and Section “Explanations regarding graphical outcomes” elaborates on the physical aspects related to the obtained solution. Notably significant findings are outlined in Section “Conclusions”.

## Model features and numerical modeling

**Figure 1:**
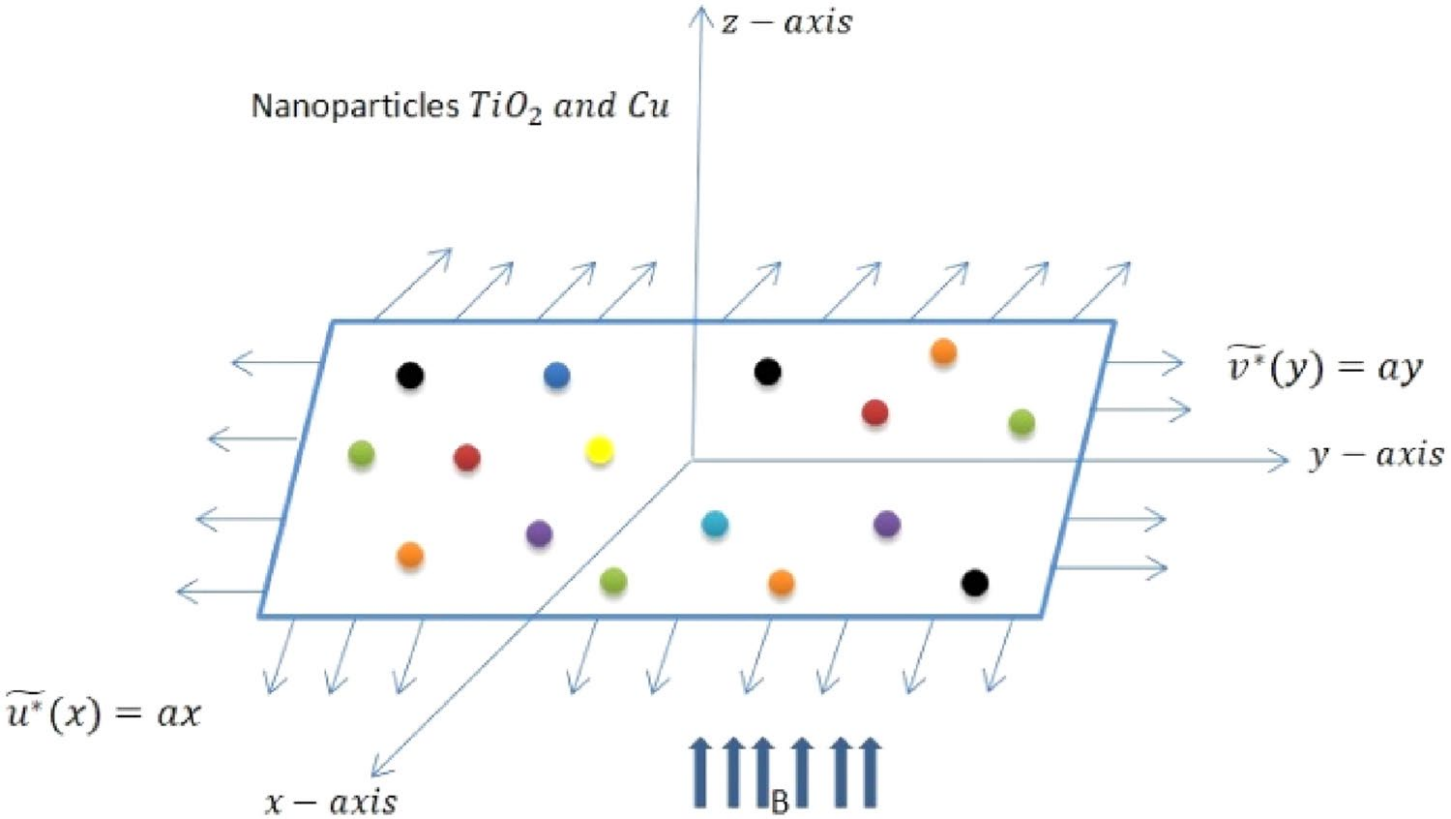
Geometry of the fluid flow occurrence with respect to coordinates.

The investigation focuses on the 3-D, incompressible boundary layer flow of titanium Titanium dioxide, Copper and Water taken as base fluids over a surface that stretches in two directions. Specifically, the study examines the scenario where the origin remains fixed and the surface experiences motion with velocities ${u}^{*}={U}_{w}\left(x\right)=ax$ and ${v}^{*}={V}_{w}(y)=by$. Here, $a$ and $b$ are positive parameters representing stretching across the $x- y$ directions as well. A magnetic field ${B}_{o}$ is applied perpendicular to the extensible surface. The underlying transport rheology is analyzed within the framework of boundary layer theory. Heat generation/ absorption, thermal radiation, and magnetic dipole are included into consideration. This physical phenomenon is illustrated in Fig. 1.

The issue that happens because of the coupled system of PDEs is

1 $$\frac{\partial \widetilde{{u}^{*}}}{\partial x}+\frac{\partial \widetilde{{v}^{*}}}{\partial y}+\frac{\partial \widetilde{{w}^{*}}}{\partial z}=0,$$

2 $$\widetilde{{u}^{*}}\frac{\partial \widetilde{{u}^{*}}}{\partial x}+\widetilde{{v}^{*}}\frac{\partial \widetilde{{u}^{*}}}{\partial y}+\widetilde{{w}^{*}}\frac{\partial \widetilde{{u}^{*}}}{\partial z}={v}_{nf}\frac{{\partial }^{2}\widetilde{{u}^{*}}}{\partial {z}^{2}}-\frac{{\sigma }_{nf}}{{\rho }_{nf}}{B}^{2}\widetilde{{u}^{*}},$$

3 $$\widetilde{{u}^{*}}\frac{\partial \widetilde{{v}^{*}}}{\partial x}+\widetilde{{v}^{*}}\frac{\partial \widetilde{{v}^{*}}}{\partial y}+\widetilde{{w}^{*}}\frac{\partial \widetilde{{v}^{*}}}{\partial z}={v}_{nf}\frac{{\partial }^{2}\widetilde{{v}^{*}}}{\partial {z}^{2}}-\frac{{\sigma }_{nf}}{{\rho }_{nf}}{B}^{2}\widetilde{{v}^{*}},$$

4 $$\widetilde{{u}^{*}}\frac{\partial T}{\partial x}+\widetilde{{v}^{*}}\frac{\partial T}{\partial y}+\widetilde{{w}^{*}}\frac{\partial T}{\partial z}={\alpha }_{nf}\frac{{\partial }^{2}T}{\partial {z}^{2}}+\frac{{\mu }_{nf}}{{\left(\rho {C}_{P}\right)}_{nf}}{\left(\frac{\partial \widetilde{{u}^{*}}}{\partial y}\right)}^{2}+\frac{Q}{{\left(\rho {C}_{P}\right)}_{nf}}\left(T-{T}_{\infty }\right)+\frac{16{\delta }^{*}{{T}_{\infty }}^{3}}{{3{K}^{*}\left({\rho C}_{P}\right)}_{nf}}\left(\frac{{\partial }^{2}T}{\partial {z}^{2}}\right).$$

Developing boundary conditions

5 $$ \begin{gathered} \widetilde{{u^{*} }} = U_{w} \left( x \right) = ax, T = T_{w} ,\widetilde{{v^{*} }} = V_{w} \left( y \right) = by, \widetilde{{w^{*} }} = 0:z = 0, \hfill \\ \widetilde{{u^{*} }} \to 0, \widetilde{{v^{*} }} \to 0, T \to T_{\infty } :z \to \infty . \hfill \\ \end{gathered} $$

The nanofluids density and viscosity are

6 $${\rho }_{nf}=\left(1-\varphi \right){\rho }_{f}+\varphi {\rho }_{s}, {\mu }_{nf}={\mu }_{f}\left(1+{B}^{1}\varphi +{B}^{2}{\varphi }^{2}\right).$$

The thermal conductivity ratio is defined as

7 $$\frac{{k}_{nf}}{{k}_{f}}=\frac{{k}_{s}+\left(m-1\right){k}_{f}+(m-1)({k}_{s}-{k}_{f})\varphi }{{k}_{s}+\left(m-1\right){k}_{f}-({k}_{s}-{k}_{f})\varphi }.$$

where $\varphi ,{k}_{nf, }{({\rho C}_{P})}_{nf}$ and ${B}^{1},{B}^{2}$ are volume friction, thermal conductivity, heat capacitance, and viscosity enhancing coefficients of the nanofluid, while ${k}_{s}, Q$ and $m$ are thermal conductivity, heat generation and form factor nanoparticles, respectively.

Similarity variables have been defined as^46^

8 $$\widetilde{{u}^{*}}=ax{f}{\prime}\left(\eta \right), \widetilde{{v}^{*}}=by{g}{\prime}\left(\eta \right), \widetilde{{w}^{*}}=-\sqrt{a{v}_{f}}\left(f\left(\eta \right)+cg\left(\eta \right)\right), \eta =\sqrt{\frac{a}{{v}_{f}}}z,\uptheta \left(\upeta \right)=\frac{{T-T}_{\infty }}{{T}_{w}-{T}_{\infty }}, a=\frac{b}{c}.$$

Dimensionless reprsentations of ODEs which are

9 $${\varepsilon }_{1}{f}^{{\prime}{\prime}{\prime}}+\left(f+ag\right){f}^{{\prime}{\prime}}-{\left({f}{\prime}\right)}^{2}-M{\varepsilon }_{3}{f}{\prime}=0,$$

10 $${\varepsilon }_{1}{g}^{{\prime}{\prime}{\prime}}+\left(f+ag\right){g}^{{\prime}{\prime}}-a{\left({g}{\prime}\right)}^{2}-M{\varepsilon }_{3}{g}{\prime}=0,$$

11 $${{(\varepsilon }_{2}+\mathrm{Rd})\uptheta }^{\boldsymbol{^{\prime}}\boldsymbol{^{\prime}}}+Pr\left(f+ag\right){\theta }{\prime}+Pr\lambda \theta +PrEc{\left({f}^{{\prime}{\prime}}\right)}^{2}=0.$$

The associations based on nanoparticles are noted below. Table 1 includes a list of the features of nanoparticles, and Table 2 includes details on the shapes of nanoparticles.

**Table 1:** Thermal characteristics related to copper and titanium within the base liquid.

Nanoparticles/base fluid	$\mathrm{Cu}$	${\mathrm{TiO}}_{2}$	${\mathrm{H}}_{2}\mathrm{O}$
$\rho$	8933	4250	997.1
${C}_{P}$	385	686.2	4179
$k$	401	8.9538	0.613
$\sigma$	59.6	0.125	5.5

**Table 2:** Shapes of nanoparticles assocaited with sizes.

Shape of nanoparticles	Shape factors (m)	${B}^{1}$	${B}^{2}$
Platelet	$5.72$	$37.1$	$612.6$
Cylinder	$4.82$	$13.5$	$904.4$
Brick	$3.72$	$1.9$	$471.4$
Sphere	$3.0$	$2.5$	$6.5$
Blade	8.26	14.6	123.3

BCS (boundary conditions) are

12 $$ \begin{gathered} f\left( 0 \right) = 0, f^{\prime}\left( 0 \right) = 1, g\left( 0 \right) = 0, g^{\prime } \left( 0 \right) = a,\theta \left( 0 \right) = 1, :\eta = 0, \hfill \\ f^{\prime}\left( \infty \right) \to 0, g^{\prime}\left( \infty \right) \to 0,\theta \left( \infty \right) \to o:\eta \to \infty . \hfill \\ \end{gathered} $$

Parametrs are defiend as

13 $$ M = \frac{{B_{0}^{2} \sigma_{f} }}{{\rho_{nf} a}},Ec = \frac{{U^{2} }}{{C_{P} \left( {T_{s} - T} \right)}}, Pr = \frac{\nu }{{\alpha_{nf} }}, \lambda = \frac{Q}{{a\left( {\rho C_{P} } \right)_{f} }}, Rd = \frac{{16\delta^{*} T_{\infty }^{3} }}{{3K^{*} \left( k \right)_{f} }}\;{\text{and}}\; a = \frac{b}{c}. $$

Now ${\varepsilon }_{1}, {\varepsilon }_{2}$ and ${\varepsilon }_{3}$ are constants which are defined as^46^

$${\varepsilon }_{1}=\frac{(1+{B}^{1}\varphi +{B}^{2}{\varphi }^{2})}{(1-\varphi +\varphi \frac{{\rho }_{s}}{{\rho }_{f}})},{\varepsilon }_{2}=\frac{\frac{{K}_{nf}}{K}}{1-\varphi +\varphi \frac{{(\rho {C}_{P})}_{s}}{{(\rho {C}_{P})}_{f}}}, {\varepsilon }_{3}=\frac{(1-\varphi +\varphi \frac{{\sigma }_{s}}{{\sigma }_{f}})}{(1-\varphi +\varphi \frac{{\rho }_{s}}{{\rho }_{f}})} .$$

Dimensionless expressions for the local nusselt number and the skin-friction coefficient, respectively.

14 $$ \begin{gathered} C_{fx} = \frac{{2\mu_{nf} \left( {\frac{\partial u}{{\partial z}}} \right)_{z = 0} }}{{\rho_{f} U^{2} }} = {\text{Re}}^{{ - \frac{1}{2}}} \left( {1 + B^{1} \varphi + B^{2} \varphi } \right)f^{\prime\prime}\left( 0 \right), \hfill \\ C_{fy} = \frac{{2\mu_{nf} \left( {\frac{\partial v}{{\partial z}}} \right)_{z = 0} }}{{\rho_{f} U^{2} }} = {\text{Re}}^{{ - \frac{1}{2}}} \left( {1 + B^{1} \varphi + B^{2} \varphi^{2} } \right)g^{\prime\prime}\left( 0 \right), \hfill \\ {\text{Nu}} = \frac{{ - xK_{nf} \left( {\frac{\partial T}{{\partial z}}} \right)_{z = 0} }}{{T_{s} - T_{0} }} = {\text{Re}}^{1/2} \frac{{K_{nf} }}{K}\theta^{\prime}\left( 0 \right). \hfill \\ \end{gathered} $$

## Optimal homotopy analysis method

The transformations from the PDE system to the ODE system have been important for performance. Using the OHAM approach, the derived flow model (ODEs) incorporating BCs is resolved. Coupled and non-linear describe the ODE system that exists within boundary conditions (BCs). We try to produce convergent homotopy solutions (OHAM) using the optimal homotopy analytical approach^19^. The auxiliary linear operators and sufficient initial approximations for homotopic solutions are provided as

15 $${f}_{0}\left(\eta \right)=1-\mathrm{exp}\left(-\eta \right),$$

16 $${g}_{0}\left(\eta \right)=a-a\mathrm{exp}(-\eta ),$$

17 $${\theta }_{0}\left(\eta \right)=\mathrm{exp}\left(-\eta \right).$$

18 $$\left.{\mathcal{L}}_{f}=\frac{{d}^{3}f}{d{\eta }^{3}}-\frac{df}{d\eta }, {\mathcal{L}}_{g}=\frac{{d}^{3}g}{d{\eta }^{3}}-\frac{dg}{d\eta },{\mathcal{L}}_{\theta }=\frac{{d}^{3}\theta }{d{\eta }^{3}}-\theta .\right\}$$

The preceding linear operators meet the following criteria

19 $${\mathcal{L}}_{f}\left\{{A}_{1}^{*}+{A}_{2}^{*}\mathrm{exp}\left(\eta \right)+{A}_{3}^{*}\mathrm{exp}\left(-\eta \right)\right\}=0,$$

20 $${\mathcal{L}}_{g}\left\{{A}_{4}^{*}+{A}_{5}^{*}\mathrm{exp}\left(\eta \right)+{A}_{6}^{*}\mathrm{exp}\left(-\eta \right)\right\}=0,$$

21 $${\mathcal{L}}_{\theta }\left\{{A}_{7}^{*}\mathrm{exp}\left(\eta \right)+{A}_{8}^{*}\mathrm{exp}\left(-\eta \right)\right\}=0.$$

The above constants ${A}_{j}^{*}=(1-8)$ which can be find by using BC.

## Explanations regarding graphical outcomes

The part played by thermal dissipation, thermal radiation, heat generation and the magnetic field in a 3D, three-directional thermal energy transport phenomenon within the Newtonian fluid. The base fluid called water contains a number of different types of nanoparticles, including Blade, Brick, Cylinder, Platelets, and Sphere. This thermal energy transmission phenomenon is being studied using numerical methods. The link between velocity, temperature, and other parameters is represented graphically; the flow model is described below.

### Compare the impacts of Cu and TiO_2_ on the velocity field

**Figure 2:**
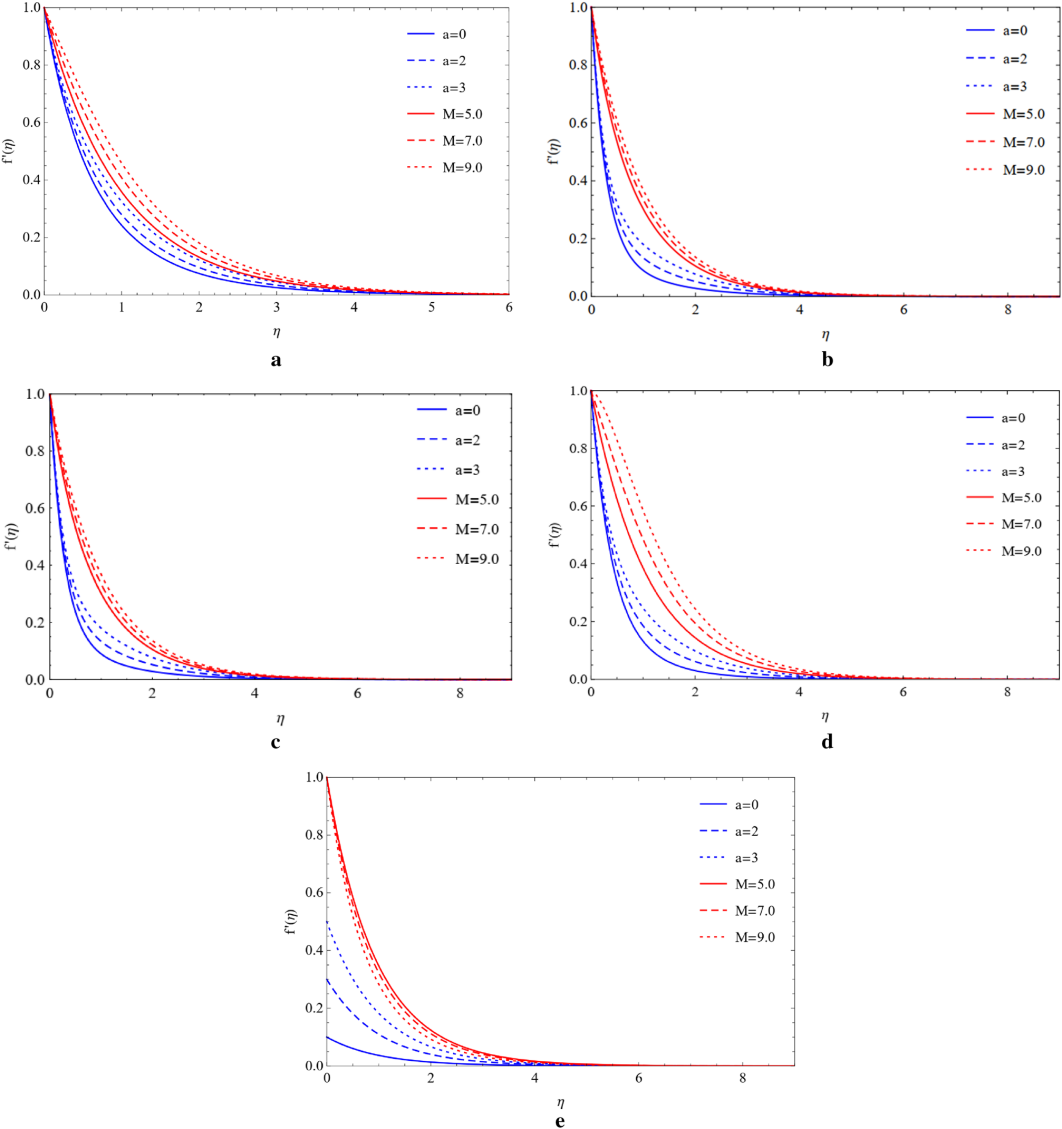
(**a**) Effect on blade of $a$ and $M$ by fixing $\mathrm{Rd}=0.1,\lambda =0.01,\mathrm{Ec}=1.0,\mathrm{Pr}=4.0,\varphi =0.5.$ (**b**) Effect on brick of $a$ and $M$ by fixing $Rd=0.1,\lambda =0.01,\mathrm{Ec}=1.0,\mathrm{Pr}=4.0,\varphi =0.5.$ (**c**) Effect on cylinder of $a$ and $M$ by fixing $Rd=0.1,\lambda =0.01,\mathrm{Ec}=1.0,\mathrm{Pr}=4.0,\varphi =0.5.$ (**d**) Effect on platelet of $a$ and $M$ by fixing $Rd=0.1,\lambda =0.01,\mathrm{Ec}=1.0,\mathrm{Pr}=4.0,\varphi =0.5.$ (**e**) Effect on sphere of $a$ and $M$ by fixing $Rd=0.1,\lambda =0.01,\mathrm{Ec}=1.0,\mathrm{Pr}=4.0,\varphi =0.5.$

**Figure 3:**
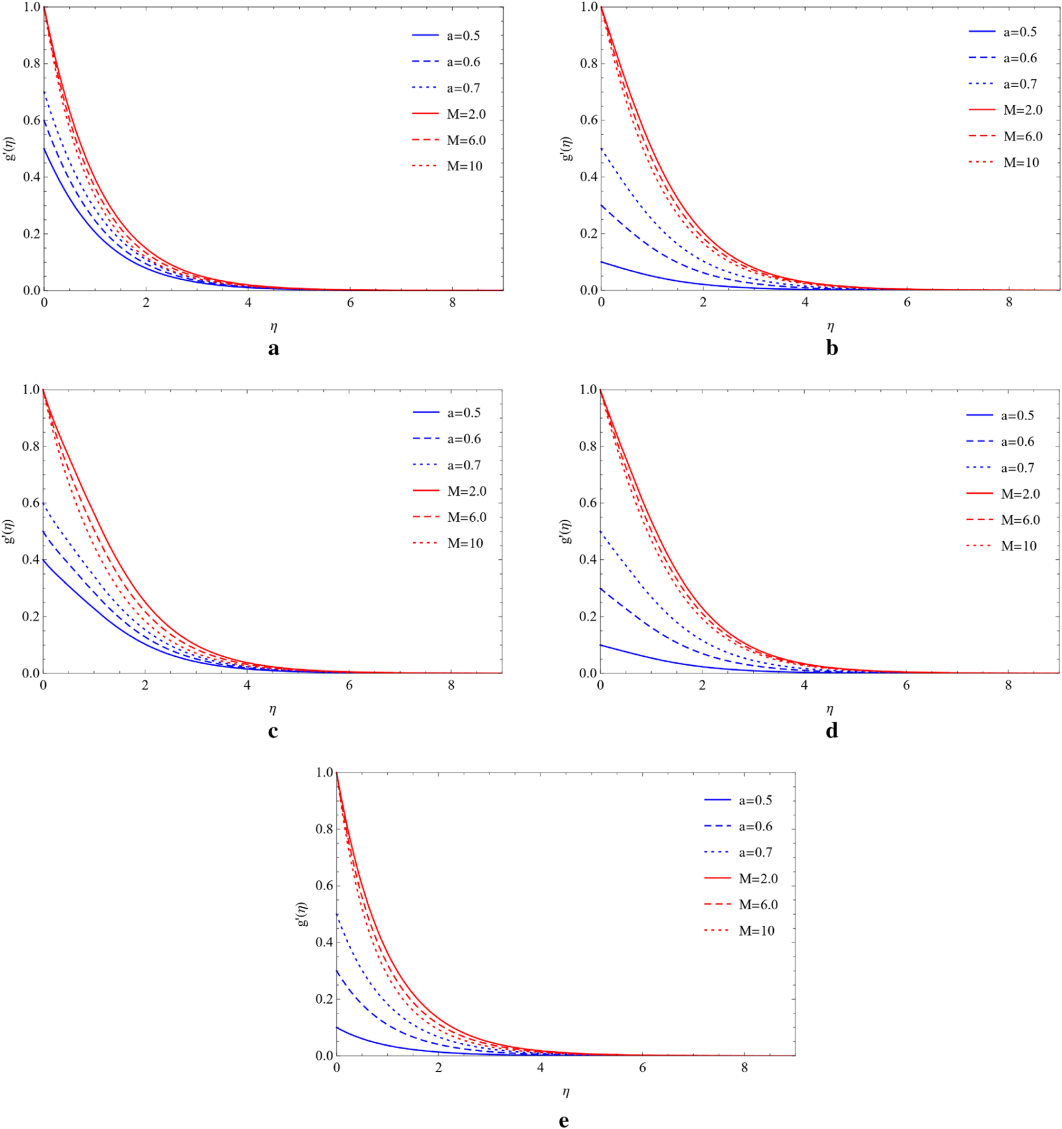
(**a**) Impact on blade of $a$ and $M$ by fixing $Rd=0.1,\lambda =0.01,\mathrm{Ec}=1.0,\mathrm{Pr}=4.0,\varphi =0.5.$ (**b**) Impact on brick of $a$ and $M$ by fixing $Rd=0.1,\lambda =0.01,\mathrm{Ec}=1.0,\mathrm{Pr}=4.0,\varphi =0.5.$ (**c**) Impact on cylinder of $a$ and $M$ by fixing $Rd=0.1,\lambda =0.01,\mathrm{Ec}=1.0,\mathrm{Pr}=4.0,\varphi =0.5.$ (**d**) Impact on platelets of $a$ and $M$ by fixing $Rd=0.1,\lambda =0.01,\mathrm{Ec}=1.0,\mathrm{Pr}=4.0,\varphi =0.5.$ (**e**) Impact on sphere of $a$ and $M$ by fixing $Rd=0.1,\lambda =0.01,\mathrm{Ec}=1.0,\mathrm{Pr}=4.0,\varphi =0.5.$

**Figure 4:**
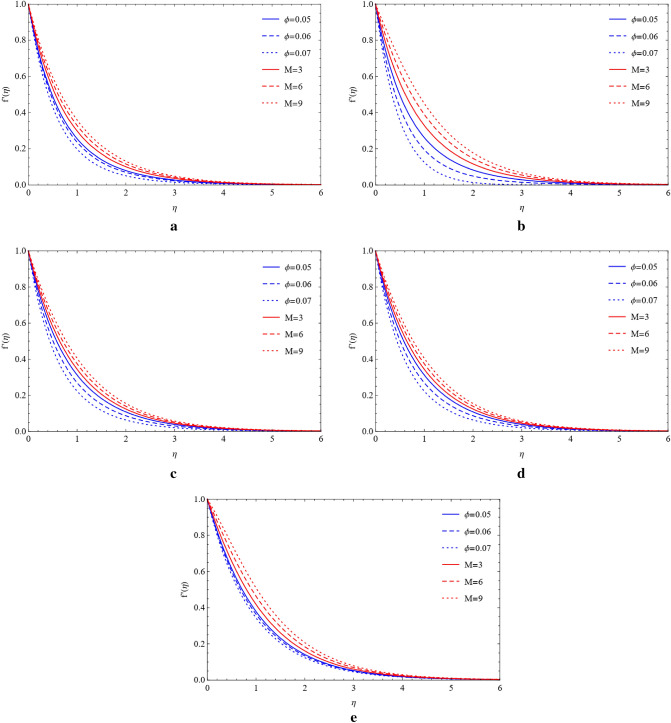
(**a**) Impact on blade of $\phi$ and $M$ by fixing $Rd=0.1,\lambda =0.01,\mathrm{Ec}=a=1.0,\mathrm{Pr}=4.0.$ (**b**) Impact on brick of $\phi$ and $M$ by fixing $Rd=0.1,\lambda =0.01,\mathrm{Ec}=a=1.0,\mathrm{Pr}=4.0.$ (**c**) Impact on cylinder of $\phi$ and $M$ by fixing $Rd=0.1,\lambda =0.01,\mathrm{Ec}=a=1.0,\mathrm{Pr}=4.0.$ (**d**) Impact on platelets of $\phi$ and $M$ by fixing $Rd=0.1,\lambda =0.01,\mathrm{Ec}=a=1.0,\mathrm{Pr}=4.0.$ (**e**) Impact on sphere of $\phi$ and $M$ by fixing $Rd=0.1,\lambda =0.01,\mathrm{Ec}=a=1.0,\mathrm{Pr}=4.0.$

**Figure 5:**
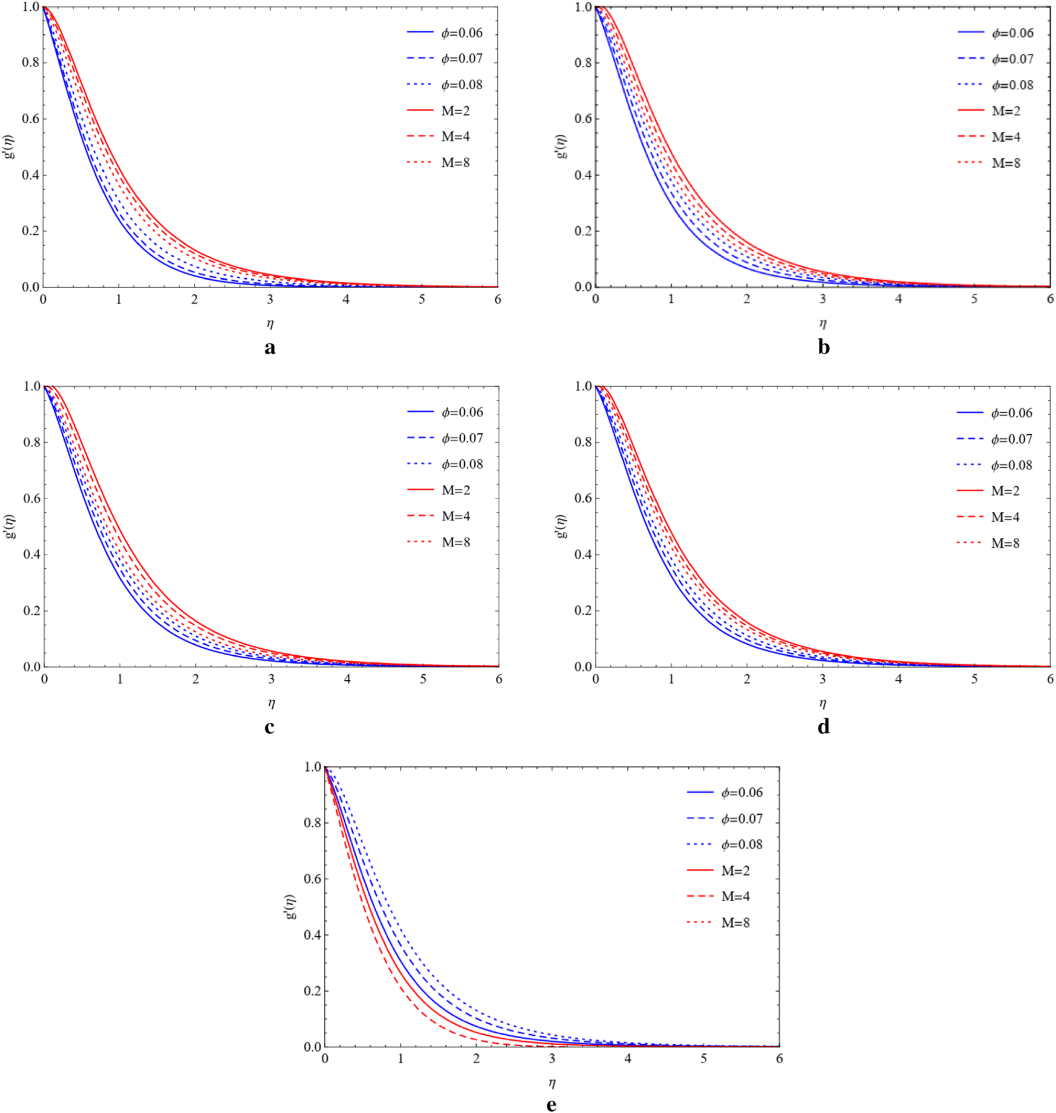
(**a**) Variation of $\phi$ and $M$ on blade by fixing $Rd=0.1,\lambda =0.01,\mathrm{Ec}=a=1.0,\mathrm{Pr}=4.0.$ (**b**) Variation of $\phi$ and $M$ on brick by fixing $Rd=0.1,\lambda =0.01,\mathrm{Ec}=a=1.0,\mathrm{Pr}=4.0.$ (**c**) Variation of $\phi$ and $M$ on cylinder by fixing $Rd=0.1,\lambda =0.01,\mathrm{Ec}=a=1.0,\mathrm{Pr}=4.0.$ (**d**) Variation of $\phi$ and $M$ on platelets by fixing $Rd=0.1,\lambda =0.01,\mathrm{Ec}=a=1.0,\mathrm{Pr}=4.0.$ (**e**) Variation of $\phi$ and $M$ on sphere by fixing $Rd=0.1,\lambda =0.01,\mathrm{Ec}=a=1.0,\mathrm{Pr}=4.0.$

Cu and TiO_2_ nanoparticles are sustained with respect to flowing phenomena when compared to the magnetic field, stretching ratio number, and volume fraction with respect to blade, brick, platelet, cylinder, and sphere-shaped nanoparticles. The influence of the magnetic number (M) and stretching ratio number (a) on the flow of copper nanoparticles in the blade, brick, platelet, sphere and cylinder forms. Figure 2a–d show how the flow increases when the magnetic number (M) and stretching ratio number (a) both rise. From Fig. 2e, it can be observed that in the presence of a sphere, the velocity field is affected by the magnetic number and ratio number. It is clearly noticed that enhancing the magnetic number the flow is slow down but in ratio number flow is opposite as compared to magnetic number. Figure 3a–e shows that when the magnetic number increases, the flow becomes slow and the stretching surface increases for the blade, brick, platelet, cylinder, and sphere types of nanoparticles the flow become high, which comprise Cu and TiO_2_. The high velocity of Cu nanoparticles in the presence of blades, bricks, platelets, cylinders, and spheres is formed by using the high value of the magnetic number (M) and the large value of volume friction ($\varphi$) generated by weak velocity, as shown in Fig. 4a–e. Figure 5a–e show the analysis of the high velocity of Cu nanoparticles in the existence of blades, bricks, platelets, cylinders, and spheres by using the big value of volume friction ($\varphi$) and the large value of magnetic number (M), which produce weak velocities.

### Compare the impacts of Cu and TiO_2_ on temperature field

**Figure 6:**
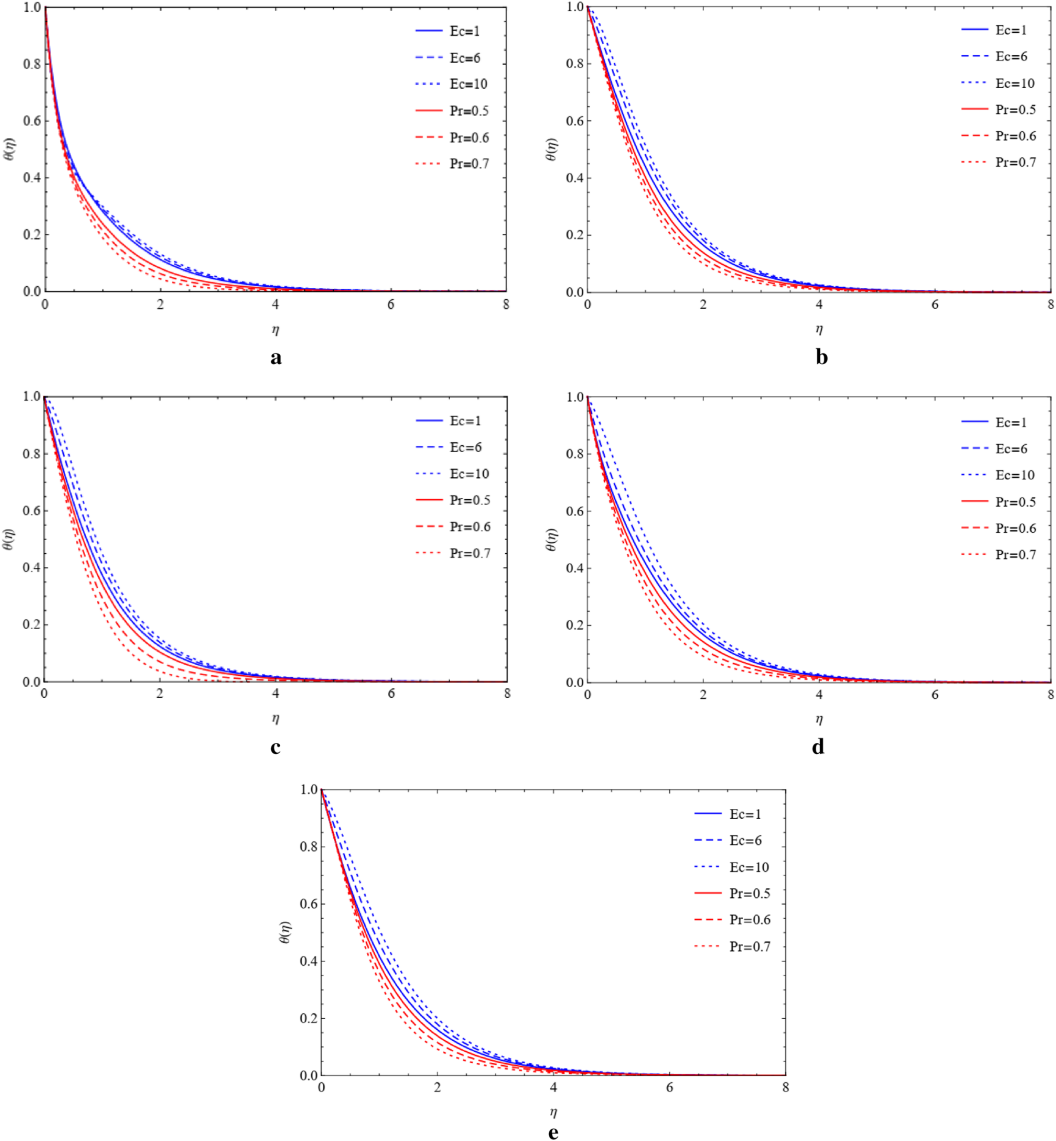
(**a**) Influence of $Ec$ and $Pr$ on blade by fixing $Rd=0.1,\lambda =0.01,a=1.0,M=0.4,\phi =0.5.$ (**b**) Influence of $Ec$ and $Pr$ on brick by fixing $Rd=0.1,\lambda =0.01,a=1.0,M=0.4,\phi =0.5.$ (**c**) Influence of $Ec$ and $Pr$ on cylinder by fixing $Rd=0.1,\lambda =0.01,a=1.0,M=0.4,\phi =0.5.$ (**d**) Influence of $Ec$ and $Pr$ on platelets by fixing $Rd=0.1,\lambda =0.01,a=1.0,M=0.4,\phi =0.5.$ (**e**) Influence of $Ec$ and $Pr$ on sphere by fixing $Rd=0.1,\lambda =0.01,a=1.0,M=0.4,\phi =0.5.$

**Figure 7:**
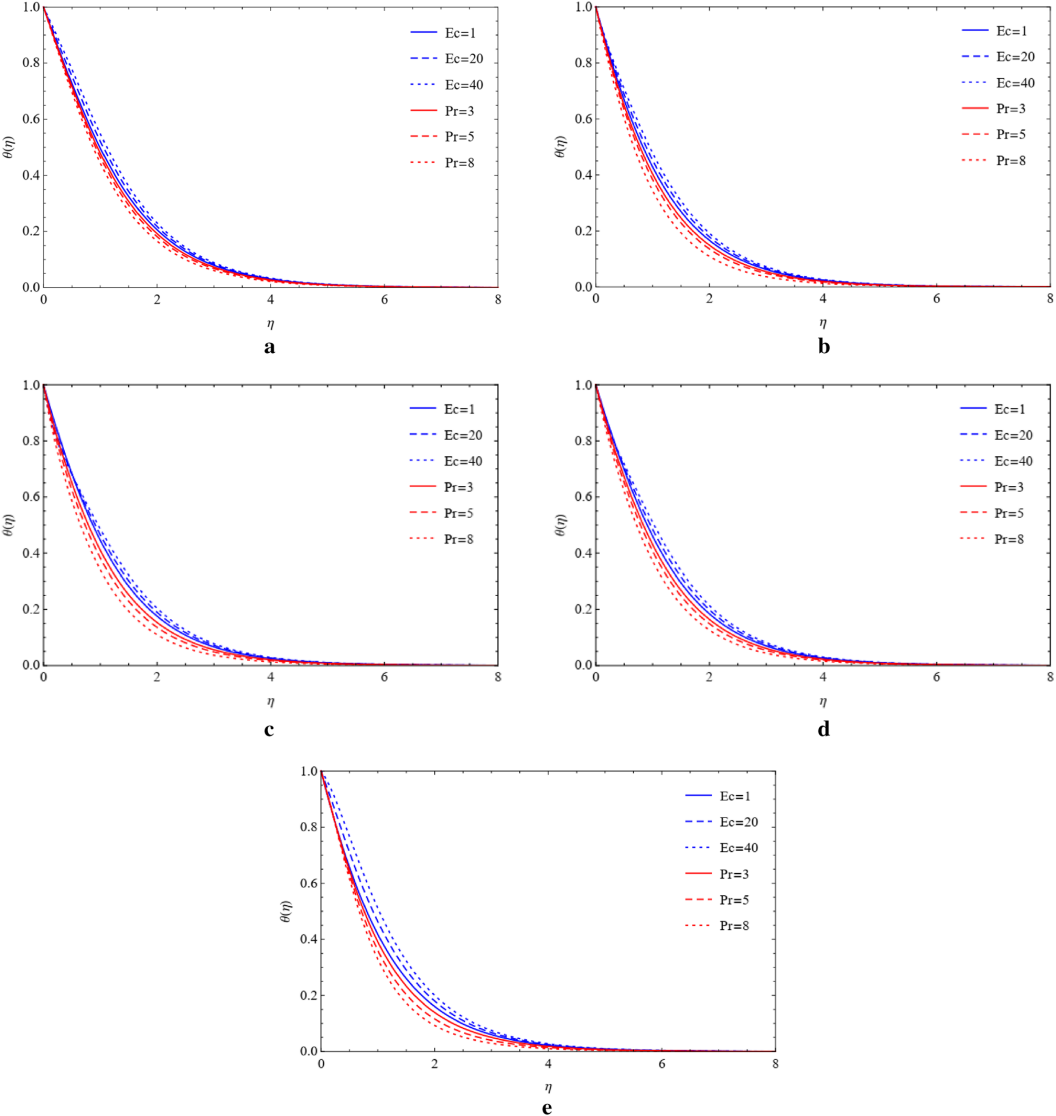
(**a**) Variation of $Ec$ and $Pr$ on blade by fixing $Rd=0.1,\lambda =0.01,a=1.0,M=0.4,\phi =0.5.$ (**b**) Variation of $Ec$ and $Pr$ on brick by fixing $Rd=0.1,\lambda =0.01,a=1.0,M=0.4,\phi =0.5.$ (**c**) Variation of $Ec$ and $Pr$ on cylinder by fixing $Rd=0.1,\lambda =0.01,a=1.0,M=0.4,\phi =0.5.$ (**d**) Variation of $Ec$ and $Pr$ on platelets by fixing $Rd=0.1,\lambda =0.01,a=1.0,M=0.4,\phi =0.5.$ (**e**) Variation of $Ec$ and $Pr$ on sphere by fixing $Rd=0.1,\lambda =0.01,a=1.0,M=0.4,\phi =0.5.$

**Figure 8:**
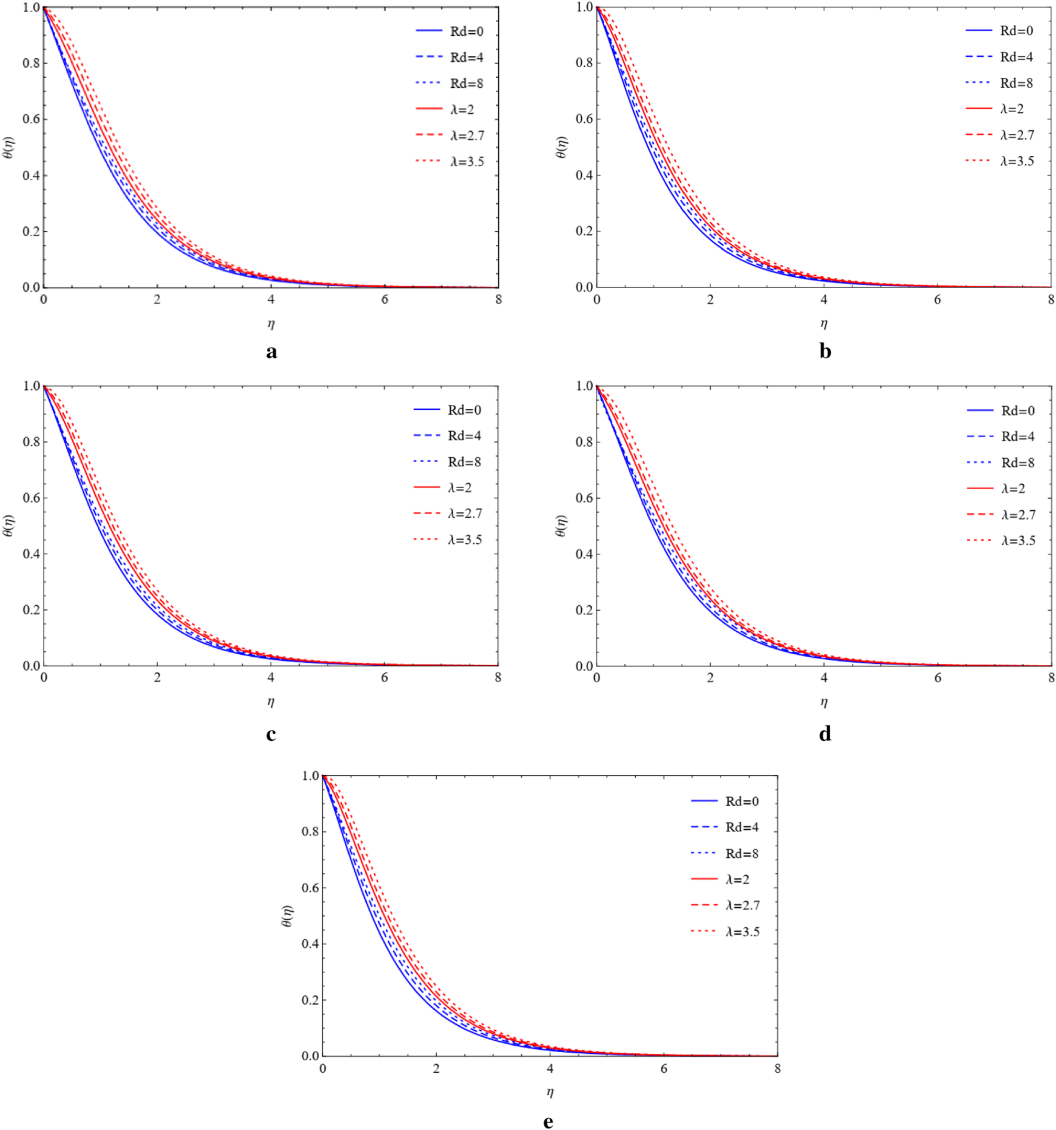
(**a**) Influence of $Rd$ and $\lambda$ on blade by fixing $\mathrm{Pr}=4.0,\mathrm{Ec}=a=1.0,M=0.4,\phi =0.5.$ (**b**) Influence of $Rd$ and $\lambda$ on brick by fixing $\mathrm{Pr}=4.0,\mathrm{Ec}=a=1.0,M=0.4,\phi =0.5.$ (**c**) Influence of $Rd$ and $\lambda$ on cylinder by fixing $\mathrm{Pr}=4.0,\mathrm{Ec}=a=1.0,M=0.4,\phi =0.5.$ (**d**) Influence of $Rd$ and $\lambda$ on platelets by fixing $\mathrm{Pr}=4.0,\mathrm{Ec}=a=1.0,M=0.4,\phi =0.5.$ (**e**) Influence of $Rd$ and $\lambda$ on sphere by fixing $\mathrm{Pr}=4.0,\mathrm{Ec}=a=1.0,M=0.4,\phi =0.5.$

**Figure 9:**
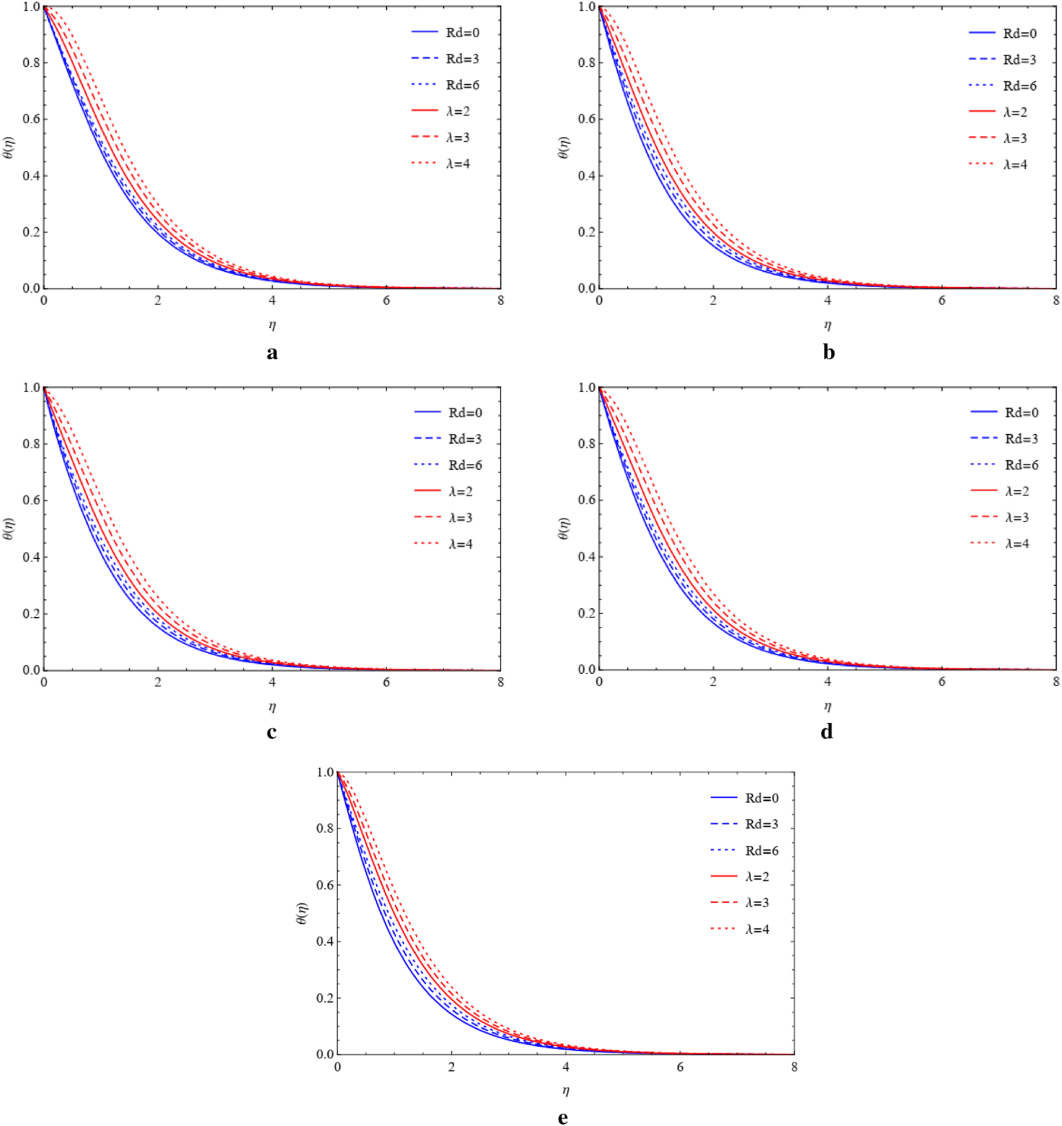
(**a**) Effect of $Rd$ and $\lambda$ on blade by fixing $\mathrm{Pr}=4.0,\mathrm{Ec}=a=1.0,M=0.4,\phi =0.5.$ (**b**) Effect of $Rd$ and $\lambda$ on brick by fixing $\mathrm{Pr}=4.0,\mathrm{Ec}=a=1.0,M=0.4,\phi =0.5.$ (**c**) Effect of $Rd$ and $\lambda$ on cylinder by fixing $\mathrm{Pr}=4.0,\mathrm{Ec}=a=1.0,M=0.4,\phi =0.5.$ (**d**) Effect of $Rd$ and $\lambda$ on platelets by fixing $\mathrm{Pr}=4.0,\mathrm{Ec}=a=1.0,M=0.4,\phi =0.5.$ (**e**) Effect of $Rd$ and $\lambda$ on sphere by fixing $\mathrm{Pr}=4.0,\mathrm{Ec}=a=1.0,M=0.4,\phi =0.5.$

The Prandtl number and Eckert number on the temperature field are estimated using the plots shown in Figs. 6a–e and 7a–e, respectively. These graphs were created to show the measurement of thermal energy in the presence of various shapes and their impacts on Cu and TiO_2_ nanoparticles. In the appearance of blade, brick, platelet, cylinder, and sphere shapes of nanoparticles, it is shown that heat energy is decreased due to the enhancing value of Prandtl and Eckert numbers that generate high heat energy. Viscous dissipation and heat energy are discovered to have a direct relationship as well. Due to this direct relationship, low heat energy is emitted since Eckert values are smaller. Figures 8a–8e and 9a–9e show the significant energy output that results from the presence of copper and titanium nanoparticles of different shapes that have high thermal radiation and heat absorption values.

## Conclusions

The OHAM algorithm has been successfully used to solve the problem of heat energy in Newtonian fluids using a dissolving 3-D surface with nanoparticles in the shapes of blades, bricks, cylinders, plates, and spheres. Important points of presented observation are listed below:

- High velocities and an increase in flow are produced by magnetic number (M) values;
- Wall momentum diffuses more slowly when the magnetic number (M) is high, but it diffuses more quickly when the stretching ratio (a) is high;
- Increasing the volume friction ($\varphi$) generate weak velocity;
- Due to the high level of Prandtl number (Pr), there is a reduction in thermal energy;
- Maximum production of heat energy due to high value of Eckert number (Ec);
- Temperature distribution is influenced by thermal radiation (Rd) and heat absorption ($\lambda$).

## Data Availability

The datasets used and/or analyzed during the current study available from the corresponding author on reasonable request.

## Abbreviations

$$\widetilde{{u}^{*}},\widetilde{{v}^{*}},\widetilde{{w}^{*}}$$: Velocity components $$({\mathrm{ms}}^{-1})$$
$a,b$: Stretching parameters $({\mathrm{S}}^{-1})$ ratio parameter
$$c$$: Ratio parameter
$${\nu }_{nf}$$: Kinematic viscosity $$({\mathrm{m}}^{2}{\mathrm{s}}^{-1})$$
$${\sigma }_{nf}$$: Electricity conductivity $$({\Omega }^{-1}{\mathrm{m}}^{-1})$$
$${\rho }_{nf}$$: Density $$({\mathrm{kgm}}^{-3})$$
$B$: Magnetic field strength $({{\Omega }^{1/2}\mathrm{m}}^{-1}{\mathrm{s}}^{-1/2}{\mathrm{kg}}^{1/2})$
$$\frac{{K}_{nf}}{K}$$: Thermal conductivity $${(\mathrm{Wm}}^{-1}{\mathrm{K}}^{-1})$$
$${\rho C}_{P}$$: Heat capacitance $$({\mathrm{Jm}}^{-3}{\mathrm{K}}^{-1})$$
$$\varphi $$: Volume friction
$m$: Shape factor nanoparticles
$$a$$: Stretching velocity $$({\mathrm{s}}^{-1})$$
$Rd$: Thermal radiation parameter $({\mathrm{Jm}}^{-2}{\mathrm{s}}^{-1})$
$$Q$$: Heat generation
${\delta }^{*}$: Stefan Boltzmann constant ${(\mathrm{Wm}}^{-2}{\mathrm{K}}^{-4})$
OHAM: Optimal homotopy analysis method
thnf: Ternary hybrid nanofluid
PEDs: Partial differential equation
$$x,y,z$$: Cartesian coordinate
${\varepsilon }_{1},{\varepsilon }_{2},{\varepsilon }_{3}$: Temperature difference parameter, concentration difference and diffusion parameter
${B}^{1},{B}^{2}$: Viscosity enhancement coefficient
$$\mathrm{Pr}$$: Prandtl number
$$M$$: Magnetic parameter
$${\mathrm{Nu}}^{*}$$: Nusselt number
$$\mathrm{Ec}$$: Eckert number
$${C}_{fx},{C}_{fy}$$: Skin friction
${k}_{s}$: Thermal conductivity nanoparticle ${(\mathrm{Wm}}^{-1}{\mathrm{K}}^{-1})$
$${T}_{s}$$: Reference temperature $$\left(\mathrm{K}\right)$$
$$T$$: Temperature nanofluid $$\left(\mathrm{K}\right)$$
$$U$$: Velocity $$\left({\mathrm{ms}}^{-1}\right)$$
$\lambda$: Heat generation parameter
$${T}_{\infty }$$: Ambient temperature $$\left(\mathrm{K}\right)$$
${K}^{*}$: Mean absorption coefficient ${(\mathrm{m}}^{-1})$
MHD: Magneto-hydrodynamic
hnf: Hybrid nanofluid
ODEs: Ordinary differential equation
